# Supplementary material for: Molecular Topology for the Discovery of New Broad-Spectrum Antibacterial Drugs
Source: Biomolecules. 2020 Sep 19;10(9):1343. doi: 10.3390/biom10091343 (PMC7564208; doi:10.3390/biom10091343)
Supplement: Supplementary file 1 [file biomolecules-10-01343-s001.zip › Table S1.doc]

**Table S1. Active group used in the topological pattern: Paper name/code, IUPAC name & structure and bibliograph**ic references about activity for each compound.

| **Paper name/code** | **IUPAC name & structure** | **References** |
| --- | --- | --- |
| A-80556 |   7-(4-amino-2-methylpyrrolidin-1-yl)-1-(2,4-difluorophenyl)-6-fluoro-4-oxo-1,4-dihydroquinoline-3-carboxylic acid | [Clement, J.J](http://www.ncbi.nlm.nih.gov/pubmed?term=Clement JJ%5BAuthor%5D&cauthor=true&cauthor_uid=8067740).; [Tanaka, S.K](http://www.ncbi.nlm.nih.gov/pubmed?term=Tanaka SK%5BAuthor%5D&cauthor=true&cauthor_uid=8067740).; [Alder, J](http://www.ncbi.nlm.nih.gov/pubmed?term=Alder J%5BAuthor%5D&cauthor=true&cauthor_uid=8067740).; [Vojtko, C](http://www.ncbi.nlm.nih.gov/pubmed?term=Vojtko C%5BAuthor%5D&cauthor=true&cauthor_uid=8067740).; [Beyer, J](http://www.ncbi.nlm.nih.gov/pubmed?term=Beyer J%5BAuthor%5D&cauthor=true&cauthor_uid=8067740).; [Hensey, D](http://www.ncbi.nlm.nih.gov/pubmed?term=Hensey D%5BAuthor%5D&cauthor=true&cauthor_uid=8067740).; [Ramer, N](http://www.ncbi.nlm.nih.gov/pubmed?term=Ramer N%5BAuthor%5D&cauthor=true&cauthor_uid=8067740).; [McDaniel, D](http://www.ncbi.nlm.nih.gov/pubmed?term=McDaniel D%5BAuthor%5D&cauthor=true&cauthor_uid=8067740).; [Chu, D.T](http://www.ncbi.nlm.nih.gov/pubmed?term=Chu DT%5BAuthor%5D&cauthor=true&cauthor_uid=8067740). *In vitro* and *in vivo* evaluations of A-80556, a new fluoroquinolone. [*Antimicrob. Agents Chemother.*](http://www.ncbi.nlm.nih.gov/pubmed/8067740), **1994**, *38*(5), 1071-1078. |
| ABT-492 |   1-(6-amino-3,5-difluoropyridin-2-yl)-8-chloro-6-fluoro-7-(3-hydroxyazetidin-1-yl)-4-oxo-1,4-dihydroquinoline-3-carboxylic acid | [Almer, L.S](http://www.ncbi.nlm.nih.gov/pubmed?term=Almer LS%5BAuthor%5D&cauthor=true&cauthor_uid=15215148).; [Hoffrage, J.B](http://www.ncbi.nlm.nih.gov/pubmed?term=Hoffrage JB%5BAuthor%5D&cauthor=true&cauthor_uid=15215148).; [Keller, E.L](http://www.ncbi.nlm.nih.gov/pubmed?term=Keller EL%5BAuthor%5D&cauthor=true&cauthor_uid=15215148).; [Flamm, R.K](http://www.ncbi.nlm.nih.gov/pubmed?term=Flamm RK%5BAuthor%5D&cauthor=true&cauthor_uid=15215148).; [Shortridge, V.D](http://www.ncbi.nlm.nih.gov/pubmed?term=Shortridge VD%5BAuthor%5D&cauthor=true&cauthor_uid=15215148). *In vitro* and bactericidal activities of ABT-492, a novel fluoroquinolone, against gram-positive and gram-negative organisms. [*Antimicrob. Agents Chemother.*](http://www.ncbi.nlm.nih.gov/pubmed/15215148), **2004**, *48*(7), 2771-2777. |
| Amifloxacin |   6-fluoro-1-(methylamino)-7-(4-methylpiperazin-1-yl)-4-oxo-1,4-dihydroquinoline-3-carboxylic acid | [Venezia, R.A](http://www.ncbi.nlm.nih.gov/pubmed?term=Venezia RA%5BAuthor%5D&cauthor=true&cauthor_uid=2751287).; [Prymas, L.A](http://www.ncbi.nlm.nih.gov/pubmed?term=Prymas LA%5BAuthor%5D&cauthor=true&cauthor_uid=2751287).; [Shayegani, A](http://www.ncbi.nlm.nih.gov/pubmed?term=Shayegani A%5BAuthor%5D&cauthor=true&cauthor_uid=2751287).; [Yocum, D.M](http://www.ncbi.nlm.nih.gov/pubmed?term=Yocum DM%5BAuthor%5D&cauthor=true&cauthor_uid=2751287). *In vitro* activities of amifloxacin and two of its metabolites. [*Antimicrob. Agents Chemother.*](http://www.ncbi.nlm.nih.gov/pubmed/2751287), **1989**, *33*(5), 762-766. |
| Balofloxacin |   1-cyclopropyl-6-fluoro-8-methoxy-7-(3-(methylamino)piperidin-1-yl)-4-oxo-1,4-dihydroquinoline-3-carboxylic acid | [Ito, T](http://www.ncbi.nlm.nih.gov/pubmed?term=Ito T%5BAuthor%5D&cauthor=true&cauthor_uid=1329626).; [Otsuki, M](http://www.ncbi.nlm.nih.gov/pubmed?term=Otsuki M%5BAuthor%5D&cauthor=true&cauthor_uid=1329626).; [Nishino, T](http://www.ncbi.nlm.nih.gov/pubmed?term=Nishino T%5BAuthor%5D&cauthor=true&cauthor_uid=1329626). *In vitro* antibacterial activity of Q-35, a new fluoroquinolone. [*Antimicrob. Agents Chemother.*](http://www.ncbi.nlm.nih.gov/pubmed/1329626), **1992**, *36*(8), 1708-1714. |
| BAY Y3118 |   8-chloro-1-cyclopropyl-6-fluoro-7-((4aS,7aS)-hexahydro-1H-pyrrolo[3,4-b]pyridin-6(2H)-yl)-4-oxo-1,4-dihydroquinoline-3-carboxylic acid | Bongaerts, G.P.A.; Hoogkamp, J.A.A. *In vitro* activities of BAY Y3118, ciprofloxacin, ofloxacin, and fleroxacin against gram-positive and gram-negative pathogens from respiratory tract and soft tissue infections. *Antimicrob. Agents Chemother.*, **1993**, *37*(9), 2017-2019. |
| BMS-340278 |   (R)-1-cyclopropyl-7-(1-methylisoindolin-5-yl)-4-oxo-1,4-dihydroquinoline-3-carboxylic acid | [Lawrence, L.E](http://www.ncbi.nlm.nih.gov/pubmed?term=Lawrence LE%5BAuthor%5D&cauthor=true&cauthor_uid=11481288).; [Wu, P](http://www.ncbi.nlm.nih.gov/pubmed?term=Wu P%5BAuthor%5D&cauthor=true&cauthor_uid=11481288).; [Fan, L](http://www.ncbi.nlm.nih.gov/pubmed?term=Fan L%5BAuthor%5D&cauthor=true&cauthor_uid=11481288).; [Gouveia, K.E](http://www.ncbi.nlm.nih.gov/pubmed?term=Gouveia KE%5BAuthor%5D&cauthor=true&cauthor_uid=11481288).; [Card, A](http://www.ncbi.nlm.nih.gov/pubmed?term=Card A%5BAuthor%5D&cauthor=true&cauthor_uid=11481288).; [Casperson, M](http://www.ncbi.nlm.nih.gov/pubmed?term=Casperson M%5BAuthor%5D&cauthor=true&cauthor_uid=11481288).; [Denbleyker, K](http://www.ncbi.nlm.nih.gov/pubmed?term=Denbleyker K%5BAuthor%5D&cauthor=true&cauthor_uid=11481288).; [Barrett, J.F](http://www.ncbi.nlm.nih.gov/pubmed?term=Barrett JF%5BAuthor%5D&cauthor=true&cauthor_uid=11481288). The inhibition and selectivity of bacterial topoisomerases by BMS-284756 and its analogues.  *J. Antimicrob. Chemother.*, **2001**, *48*(2), 195-201. |
| BMS-340280 |   (R)-1-cyclopropyl-8-(difluoromethoxy)-6-fluoro-7-(1-methylisoindolin-5-yl)-4-oxo-1,4-dihydroquinoline-3-carboxylic acid | Same as for BMS-340278 |
| BMS-433366 |   (R)-1-cyclopropyl-8-methoxy-7-(1-methylisoindolin-5-yl)-4-oxo-1,4-dihydroquinoline-3-carboxylic acid | Same as for BMS-340278 |
| CFC-222 |   7-((1R,6S)-6-amino-1-methyl-3-azabicyclo[3.2.0]heptan-3-yl)-1-cyclopropyl-6-fluoro-4-oxo-1,4-dihydro-1,8-naphthyridine-3-carboxylic acid | [Kim, J.H](http://www.ncbi.nlm.nih.gov/pubmed?term=Kim JH%5BAuthor%5D&cauthor=true&cauthor_uid=9533464).; [Choi, K.H](http://www.ncbi.nlm.nih.gov/pubmed?term=Choi KH%5BAuthor%5D&cauthor=true&cauthor_uid=9533464).; [Kim, J.W](http://www.ncbi.nlm.nih.gov/pubmed?term=Kim JW%5BAuthor%5D&cauthor=true&cauthor_uid=9533464).; [Lee, J.H](http://www.ncbi.nlm.nih.gov/pubmed?term=Lee JH%5BAuthor%5D&cauthor=true&cauthor_uid=9533464).; [Choi, E.C](http://www.ncbi.nlm.nih.gov/pubmed?term=Choi EC%5BAuthor%5D&cauthor=true&cauthor_uid=9533464).; [Kim, B.K](http://www.ncbi.nlm.nih.gov/pubmed?term=Kim BK%5BAuthor%5D&cauthor=true&cauthor_uid=9533464). *In-vitro* and *in-vivo* antibacterial activity of CFC-222, a new fluoroquinolone. [*J. Antimicrob. Chemother.*](http://www.ncbi.nlm.nih.gov/pubmed/9533464), **1998**, *41*(2), 223-229. |
| Ciprofloxacin |   1-cyclopropyl-6-fluoro-4-oxo-7-(piperazin-1-yl)-1,4-dihydroquinoline-3-carboxylic acid | Same as for ABT-492 |
| Clinafloxacin |   7-(3-aminopyrrolidin-1-yl)-8-chloro-1-cyclopropyl-6-fluoro-4-oxo-1,4-dihydroquinoline-3-carboxylic acid | Milatovic, D.; Schmitz, F.J.; Brisse, S.; Verhoef, J.; Fluit, A.C. *In vitro* activities of sitafloxacin (DU-6859a) and six other fluoroquinolones against 8,796 clinical bacterial isolates. *Antimicrob. Agents Chemother.*, **2000**, *44*(4), 1102-1107. |
| CS-940 |   (S)-1-cyclopropyl-8-(difluoromethoxy)-6-fluoro-7-(3-methylpiperazin-1-yl)-4-oxo-1,4-dihydroquinoline-3-carboxylic acid | [Biedenbach, D.J](http://www.ncbi.nlm.nih.gov/pubmed?term=Biedenbach DJ%5BAuthor%5D&cauthor=true&cauthor_uid=8619590).; [Sutton, L.D](http://www.ncbi.nlm.nih.gov/pubmed?term=Sutton LD%5BAuthor%5D&cauthor=true&cauthor_uid=8619590).; [Jones, R.N](http://www.ncbi.nlm.nih.gov/pubmed?term=Jones RN%5BAuthor%5D&cauthor=true&cauthor_uid=8619590). Antimicrobial activity of CS-940, a new trifluorinated quinolone. [*Antimicrob. Agents Chemother.*](http://www.ncbi.nlm.nih.gov/pubmed/?term=Antimicrobial+activity+of+CS-940%2C+a+new+trifluorinated+quinolone), **1995**, *39*(10), 2325-2330. |
| Difloxacin |   6-fluoro-1-(4-fluorophenyl)-7-(4-methylpiperazin-1-yl)-4-oxo-1,4-dihydroquinoline-3-carboxylic acid | [Stamm, J.M](http://www.ncbi.nlm.nih.gov/pubmed?term=Stamm JM%5BAuthor%5D&cauthor=true&cauthor_uid=3087274).; [Hanson, C.W](http://www.ncbi.nlm.nih.gov/pubmed?term=Hanson CW%5BAuthor%5D&cauthor=true&cauthor_uid=3087274).; [Chu, D.T](http://www.ncbi.nlm.nih.gov/pubmed?term=Chu DT%5BAuthor%5D&cauthor=true&cauthor_uid=3087274).; [Bailer, R](http://www.ncbi.nlm.nih.gov/pubmed?term=Bailer R%5BAuthor%5D&cauthor=true&cauthor_uid=3087274).; [Vojtko, C](http://www.ncbi.nlm.nih.gov/pubmed?term=Vojtko C%5BAuthor%5D&cauthor=true&cauthor_uid=3087274).; [Fernandes, P.B](http://www.ncbi.nlm.nih.gov/pubmed?term=Fernandes PB%5BAuthor%5D&cauthor=true&cauthor_uid=3087274). *In vitro* evaluation of A-56619 (difloxacin) and A-56620: new aryl-fluoroquinolones. [*Antimicrob. Agents Chemother.*](http://www.ncbi.nlm.nih.gov/pubmed/?term=J.+M.+Stamm+et+al.%2C+Antimicrob.+Ag.+Chemother.+29%2C+193+(1986)), **1986**, *29*(2), 193-200. |
| DK-507k |   7-((S)-7-amino-5-azaspiro[2.4]heptan-5-yl)-6-fluoro-1-((1R,2S)-2-fluorocyclopropyl)-8-methoxy-4-oxo-1,4-dihydroquinoline-3-carboxylic acid | [Otani, T](http://www.ncbi.nlm.nih.gov/pubmed?term=Otani T%5BAuthor%5D&cauthor=true&cauthor_uid=14638477).; [Tanaka, M](http://www.ncbi.nlm.nih.gov/pubmed?term=Tanaka M%5BAuthor%5D&cauthor=true&cauthor_uid=14638477).; [Ito, E](http://www.ncbi.nlm.nih.gov/pubmed?term=Ito E%5BAuthor%5D&cauthor=true&cauthor_uid=14638477).; [Kurosaka, Y](http://www.ncbi.nlm.nih.gov/pubmed?term=Kurosaka Y%5BAuthor%5D&cauthor=true&cauthor_uid=14638477).; [Murakami, Y](http://www.ncbi.nlm.nih.gov/pubmed?term=Murakami Y%5BAuthor%5D&cauthor=true&cauthor_uid=14638477).; [Onodera, K](http://www.ncbi.nlm.nih.gov/pubmed?term=Onodera K%5BAuthor%5D&cauthor=true&cauthor_uid=14638477).; [Akasaka, T](http://www.ncbi.nlm.nih.gov/pubmed?term=Akasaka T%5BAuthor%5D&cauthor=true&cauthor_uid=14638477).; [Sato, K](http://www.ncbi.nlm.nih.gov/pubmed?term=Sato K%5BAuthor%5D&cauthor=true&cauthor_uid=14638477). *In vitro* and *in vivo* antibacterial activities of DK-507k, a novel fluoroquinolone. [*Antimicrob. Agents Chemother.*](http://www.ncbi.nlm.nih.gov/pubmed/14638477), **2003**, *47*(12), 3750-3759. |
| DQ-113 |   5-amino-7-((3R,4S)-3-(1-aminocyclopropyl)-4-fluoropyrrolidin-1-yl)-6-fluoro-1-((1R,2S)-2-fluorocyclopropyl)-8-methyl-4-oxo-1,4-dihydroquinoline-3-carboxylic acid | [Tanaka, M](http://www.ncbi.nlm.nih.gov/pubmed?term=Tanaka M%5BAuthor%5D&cauthor=true&cauthor_uid=11850285).; [Yamazaki, E](http://www.ncbi.nlm.nih.gov/pubmed?term=Yamazaki E%5BAuthor%5D&cauthor=true&cauthor_uid=11850285).; [Chiba, M](http://www.ncbi.nlm.nih.gov/pubmed?term=Chiba M%5BAuthor%5D&cauthor=true&cauthor_uid=11850285).; [Yoshihara, K](http://www.ncbi.nlm.nih.gov/pubmed?term=Yoshihara K%5BAuthor%5D&cauthor=true&cauthor_uid=11850285).; [Akasaka, T](http://www.ncbi.nlm.nih.gov/pubmed?term=Akasaka T%5BAuthor%5D&cauthor=true&cauthor_uid=11850285).; [Takemura, M](http://www.ncbi.nlm.nih.gov/pubmed?term=Takemura M%5BAuthor%5D&cauthor=true&cauthor_uid=11850285).; [Sato, K](http://www.ncbi.nlm.nih.gov/pubmed?term=Sato K%5BAuthor%5D&cauthor=true&cauthor_uid=11850285). *In vitro* antibacterial activities of DQ-113, a potent quinolone, against clinical isolates. [*Antimicrob. Agents Chemother.*](http://www.ncbi.nlm.nih.gov/pubmed/11850285), **2002**, *46*(3), 904-908. |
| DV-7751a |   10-(8-amino-6-azaspiro[3.4]octan-6-yl)-9-fluoro-3-methyl-7-oxo-3,7-dihydro-2H-[1,4]oxazino[2,3,4-ij]quinoline-6-carboxylic acid | [Biedenbach, D.J](http://www.ncbi.nlm.nih.gov/pubmed?term=Biedenbach DJ%5BAuthor%5D&cauthor=true&cauthor_uid=8619590).; [Jones, R.N](http://www.ncbi.nlm.nih.gov/pubmed?term=Jones RN%5BAuthor%5D&cauthor=true&cauthor_uid=8619590). *In vitro* evaluation of DV-7751a, a new fluoroquinolone with an enhanced spectrum of activity against gram-positive aerobic organisms and anaerobes. [*Antimicrob. Agents Chemother.*](http://www.ncbi.nlm.nih.gov/pubmed/?term=Antimicrobial+activity+of+CS-940%2C+a+new+trifluorinated+quinolone), **1995**, *39*(7), 1636-1643. |
| DW-116 |   6-fluoro-1-(5-fluoropyridin-2-yl)-7-(4-methylpiperazin-1-yl)-4-oxo-1,4-dihydroquinoline-3-carboxylic acid | [Choi, K.H](http://www.ncbi.nlm.nih.gov/pubmed?term=Choi KH%5BAuthor%5D&cauthor=true&cauthor_uid=9145824).; [Hong, J.S](http://www.ncbi.nlm.nih.gov/pubmed?term=Hong JS%5BAuthor%5D&cauthor=true&cauthor_uid=9145824).; [Kim, S.K](http://www.ncbi.nlm.nih.gov/pubmed?term=Kim SK%5BAuthor%5D&cauthor=true&cauthor_uid=9145824).; [Lee, D.K](http://www.ncbi.nlm.nih.gov/pubmed?term=Lee DK%5BAuthor%5D&cauthor=true&cauthor_uid=9145824).; [Yoon, S.J](http://www.ncbi.nlm.nih.gov/pubmed?term=Yoon SJ%5BAuthor%5D&cauthor=true&cauthor_uid=9145824).; [Choi, E.C](http://www.ncbi.nlm.nih.gov/pubmed?term=Choi EC%5BAuthor%5D&cauthor=true&cauthor_uid=9145824). *In-vitro* and *in-vivo* activities of DW-116, a new fluoroquinolone. [*J. Antimicrob. Chemother.*](http://www.ncbi.nlm.nih.gov/pubmed/9145824), **1997**, *39*(4), 509-514. |
| DW286 |   (E)-7-(3-(aminomethyl)-4-(methoxyimino)-3-methylpyrrolidin-1-yl)-1-cyclopropyl-6-fluoro-4-oxo-1,4-dihydro-1,8-naphthyridine-3-carboxylic acid | [Yun, H.J](http://www.ncbi.nlm.nih.gov/pubmed?term=Yun HJ%5BAuthor%5D&cauthor=true&cauthor_uid=12183275).; [Min, Y.H](http://www.ncbi.nlm.nih.gov/pubmed?term=Min YH%5BAuthor%5D&cauthor=true&cauthor_uid=12183275).; [Lim, J.A](http://www.ncbi.nlm.nih.gov/pubmed?term=Lim JA%5BAuthor%5D&cauthor=true&cauthor_uid=12183275).; [Kang, J.W](http://www.ncbi.nlm.nih.gov/pubmed?term=Kang JW%5BAuthor%5D&cauthor=true&cauthor_uid=12183275).; [Kim, S.Y](http://www.ncbi.nlm.nih.gov/pubmed?term=Kim SY%5BAuthor%5D&cauthor=true&cauthor_uid=12183275).; [Kim, M.J](http://www.ncbi.nlm.nih.gov/pubmed?term=Kim MJ%5BAuthor%5D&cauthor=true&cauthor_uid=12183275).; [Jeong, J.H](http://www.ncbi.nlm.nih.gov/pubmed?term=Jeong JH%5BAuthor%5D&cauthor=true&cauthor_uid=12183275).; [Choi, Y.J](http://www.ncbi.nlm.nih.gov/pubmed?term=Choi YJ%5BAuthor%5D&cauthor=true&cauthor_uid=12183275).; [Kwon, H.J](http://www.ncbi.nlm.nih.gov/pubmed?term=Kwon HJ%5BAuthor%5D&cauthor=true&cauthor_uid=12183275).; [Jung, Y.H](http://www.ncbi.nlm.nih.gov/pubmed?term=Jung YH%5BAuthor%5D&cauthor=true&cauthor_uid=12183275).; [Shim, M.J](http://www.ncbi.nlm.nih.gov/pubmed?term=Shim MJ%5BAuthor%5D&cauthor=true&cauthor_uid=12183275).; [Choi, E.C](http://www.ncbi.nlm.nih.gov/pubmed?term=Choi EC%5BAuthor%5D&cauthor=true&cauthor_uid=12183275). *In vitro* and *in vivo* antibacterial activities of DW286, a new fluoronaphthyridone antibiotic. [*Antimicrob. Agents Chemother.*](http://www.ncbi.nlm.nih.gov/pubmed/12183275), **2002**, *46*(9), 3071-3074. |
| DX-619 |   7-((R)-3-(1-aminocyclopropyl)pyrrolidin-1-yl)-1-((1R,2S)-2-fluorocyclopropyl)-8-methoxy-4-oxo-1,4-dihydroquinoline-3-carboxylic acid | [Fujikawa, K](http://www.ncbi.nlm.nih.gov/pubmed?term=Fujikawa K%5BAuthor%5D&cauthor=true&cauthor_uid=15980395).; [Chiba, M](http://www.ncbi.nlm.nih.gov/pubmed?term=Chiba M%5BAuthor%5D&cauthor=true&cauthor_uid=15980395).; [Tanaka, M](http://www.ncbi.nlm.nih.gov/pubmed?term=Tanaka M%5BAuthor%5D&cauthor=true&cauthor_uid=15980395).; [Sato, K](http://www.ncbi.nlm.nih.gov/pubmed?term=Sato K%5BAuthor%5D&cauthor=true&cauthor_uid=15980395). *In vitro* antibacterial activity of DX-619, a novel des-fluoro(6) quinolone. *Antimicrob. Agents Chemother.*, **2005**, *49*(7), 3040-3045. |
| E-4441 |   7-(3-amino-3-methylcyclobutyl)-1-cyclopropyl-6,8-difluoro-4-oxo-1,4-dihydroquinoline-3-carboxylic acid | [Gargallo-Viola, D](http://www.ncbi.nlm.nih.gov/pubmed?term=Gargallo-Viola D%5BAuthor%5D&cauthor=true&cauthor_uid=2088187).; [Esteve, M](http://www.ncbi.nlm.nih.gov/pubmed?term=Esteve M%5BAuthor%5D&cauthor=true&cauthor_uid=2088187).; [Moros, M](http://www.ncbi.nlm.nih.gov/pubmed?term=Moros M%5BAuthor%5D&cauthor=true&cauthor_uid=2088187).; [Coll, R](http://www.ncbi.nlm.nih.gov/pubmed?term=Coll R%5BAuthor%5D&cauthor=true&cauthor_uid=2088187).; [Xicota, M.A](http://www.ncbi.nlm.nih.gov/pubmed?term=Xicota MA%5BAuthor%5D&cauthor=true&cauthor_uid=2088187).; [de Andres, C](http://www.ncbi.nlm.nih.gov/pubmed?term=de Andres C%5BAuthor%5D&cauthor=true&cauthor_uid=2088187).; [Roser, R](http://www.ncbi.nlm.nih.gov/pubmed?term=Roser R%5BAuthor%5D&cauthor=true&cauthor_uid=2088187).; [Guinea, J](http://www.ncbi.nlm.nih.gov/pubmed?term=Guinea J%5BAuthor%5D&cauthor=true&cauthor_uid=2088187). Comparative *in vitro* and *in vivo* activities of 6 new monofluoroquinolone and difluoroquinolone 3-carboxylic acids with a 7-azetidin ring substituent. *Antimicrob. Agents Chemother.*, **1990**, *34*(12), 2318-2326. |
| E-4474 |   7-(3-aminocyclobutyl)-1-cyclopropyl-6,8-difluoro-4-oxo-1,4-dihydroquinoline-3-carboxylic acid | Same as for E-4441 |
| E-4501 |   1-cyclopropyl-6,8-difluoro-7-(3-(methylamino)cyclobutyl)-4-oxo-1,4-dihydroquinoline-3-carboxylic acid | Same as for E-4441 |
| E-4534 |   7-((2R,3R)-3-amino-2-methylcyclobutyl)-1-cyclopropyl-6,8-difluoro-4-oxo-1,4-dihydroquinoline-3-carboxylic acid | [Coll, R](http://www.ncbi.nlm.nih.gov/pubmed?term=Coll R%5BAuthor%5D&cauthor=true&cauthor_uid=8787924).; [Gargallo-Viola, D](http://www.ncbi.nlm.nih.gov/pubmed?term=Gargallo-Viola D%5BAuthor%5D&cauthor=true&cauthor_uid=8787924).; [Tudela, E](http://www.ncbi.nlm.nih.gov/pubmed?term=Tudela E%5BAuthor%5D&cauthor=true&cauthor_uid=8787924).; [Xicota, M.A](http://www.ncbi.nlm.nih.gov/pubmed?term=Xicota MA%5BAuthor%5D&cauthor=true&cauthor_uid=8787924).; [Llovera, S](http://www.ncbi.nlm.nih.gov/pubmed?term=Llovera S%5BAuthor%5D&cauthor=true&cauthor_uid=8787924).; [Guinea, J](http://www.ncbi.nlm.nih.gov/pubmed?term=Guinea J%5BAuthor%5D&cauthor=true&cauthor_uid=8787924). Antibacterial activity and pharmacokinetics of four new 7-azetidinyl fluoroquinolones. *Antimicrob. Agents Chemother.*, **1996**, *40*(1), 274-277. |
| E-4535 |   7-((2R,3R)-3-amino-2-methylcyclobutyl)-1-cyclopropyl-6-fluoro-4-oxo-1,4-dihydroquinoline-3-carboxylic acid | Same as for E-4534 |
| E-4767 |   7-((2S,3R)-3-amino-2-methylazetidin-1-yl)-8-chloro-1-cyclopropyl-6-fluoro-4-oxo-1,4-dihydroquinoline-3-carboxylic acid | [Gargallo-Viola, D](http://www.ncbi.nlm.nih.gov/pubmed?term=Gargallo-Viola D%5BAuthor%5D&cauthor=true&cauthor_uid=11600365).; [Ferrer, S](http://www.ncbi.nlm.nih.gov/pubmed?term=Ferrer S%5BAuthor%5D&cauthor=true&cauthor_uid=11600365).; [Tudela, E](http://www.ncbi.nlm.nih.gov/pubmed?term=Tudela E%5BAuthor%5D&cauthor=true&cauthor_uid=11600365).; [Robert, M](http://www.ncbi.nlm.nih.gov/pubmed?term=Robert M%5BAuthor%5D&cauthor=true&cauthor_uid=11600365).; [Coll, R](http://www.ncbi.nlm.nih.gov/pubmed?term=Coll R%5BAuthor%5D&cauthor=true&cauthor_uid=11600365).; [Roser, R](http://www.ncbi.nlm.nih.gov/pubmed?term=Roser R%5BAuthor%5D&cauthor=true&cauthor_uid=11600365).; [Guinea, J](http://www.ncbi.nlm.nih.gov/pubmed?term=Guinea J%5BAuthor%5D&cauthor=true&cauthor_uid=11600365). Antibacterial activities and pharmacokinetics of E-4767 and E-5065, two new 8-chlorofluoroquinolones with a 7-azetidin ring substituent. *Antimicrob. Agents Chemother.*, **2001**, *45*(11), 3113-3121. |
| E-5065 |   7-(3-aminocyclobutyl)-8-chloro-1-cyclopropyl-6-fluoro-4-oxo-1,4-dihydroquinoline-3-carboxylic acid | Same as for E-4767 |
| Enoxacin |   1-ethyl-6-fluoro-4-oxo-7-(piperazin-1-yl)-1,4-dihydro-1,8-naphthyridine-3-carboxylic acid | [Gooding, B.B](http://www.ncbi.nlm.nih.gov/pubmed?term=Gooding BB%5BAuthor%5D&cauthor=true&cauthor_uid=8043036).; [Jones, R.N](http://www.ncbi.nlm.nih.gov/pubmed?term=Jones RN%5BAuthor%5D&cauthor=true&cauthor_uid=8043036). *In vitro* antimicrobial activity of CP-99,219, a novel azabicyclo-naphthyridone. *Antimicrob. Agents Chemother.*, **1993**, *37*(2), 349-353. |
| Fleroxacin |   6,8-difluoro-1-(2-fluoroethyl)-7-(4-methylpiperazin-1-yl)-4-oxo-1,4-dihydroquinoline-3-carboxylic acid | Same as for E-4767 |
| Garenoxacin |   (R)-1-cyclopropyl-8-(difluoromethoxy)-7-(1-methylisoindolin-5-yl)-4-oxo-1,4-dihydroquinoline-3-carboxylic acid | Same as for DK-507k |
| Gatifloxacin |   1-cyclopropyl-6-fluoro-8-methoxy-7-(3-methylpiperazin-1-yl)-4-oxo-1,4-dihydroquinoline-3-carboxylic acid | Same as for ABT-492 |
| Gemifloxacin |   (Z)-7-(3-(aminomethyl)-4-(methoxyimino)pyrrolidin-1-yl)-1-cyclopropyl-6-fluoro-4-oxo-1,4-dihydro-1,8-naphthyridine-3-carboxylic acid | Same as for DW286 |
| Grepafloxacin |   1-cyclopropyl-6-fluoro-5-methyl-7-(3-methylpiperazin-1-yl)-4-oxo-1,4-dihydroquinoline-3-carboxylic acid | [Neu, H.C](http://www.ncbi.nlm.nih.gov/pubmed?term=Neu HC%5BAuthor%5D&cauthor=true&cauthor_uid=1329620).; [Fang, W](http://www.ncbi.nlm.nih.gov/pubmed?term=Fang W%5BAuthor%5D&cauthor=true&cauthor_uid=1329620).; [Gu, J.W](http://www.ncbi.nlm.nih.gov/pubmed?term=Gu JW%5BAuthor%5D&cauthor=true&cauthor_uid=1329620).; [Chin, N.X](http://www.ncbi.nlm.nih.gov/pubmed?term=Chin NX%5BAuthor%5D&cauthor=true&cauthor_uid=1329620). *In vitro* activity of OPC-17116. [*Antimicrob. Agents Chemother.*](http://www.ncbi.nlm.nih.gov/pubmed/1329620), **1992**, *36*(6), 1310-1315. |
| Levofloxacin |   (S)-9-fluoro-3-methyl-10-(4-methylpiperazin-1-yl)-7-oxo-3,7-dihydro-2H-[1,4]oxazino[2,3,4-ij]quinoline-6-carboxylic acid | Same as for ABT-492 |
| Lomefloxacin |   1-ethyl-6,8-difluoro-7-(3-methylpiperazin-1-yl)-4-oxo-1,4-dihydroquinoline-3-carboxylic acid | Same as for A-80556 |
| Moxifloxacin |   1-cyclopropyl-6-fluoro-7-((4aR,7aR)-hexahydro-1H-pyrrolo[3,4-b]pyridin-6(2H)-yl)-8-methoxy-4-oxo-1,4-dihydroquinoline-3-carboxylic acid | Same as for ABT-492 |
| Norfloxacin |   1-ethyl-6-fluoro-4-oxo-7-(piperazin-1-yl)-1,4-dihydroquinoline-3-carboxylic acid | [Cunha, B.A](http://www.ncbi.nlm.nih.gov/pubmed?term=Cunha BA%5BAuthor%5D&cauthor=true&cauthor_uid=9222067).; [Qadri, S.M](http://www.ncbi.nlm.nih.gov/pubmed?term=Qadri SM%5BAuthor%5D&cauthor=true&cauthor_uid=9222067).; [Ueno, Y](http://www.ncbi.nlm.nih.gov/pubmed?term=Ueno Y%5BAuthor%5D&cauthor=true&cauthor_uid=9222067).; [Walters, E.A](http://www.ncbi.nlm.nih.gov/pubmed?term=Walters EA%5BAuthor%5D&cauthor=true&cauthor_uid=9222067).; [Domenico, P](http://www.ncbi.nlm.nih.gov/pubmed?term=Domenico P%5BAuthor%5D&cauthor=true&cauthor_uid=9222067). Antibacterial activity of trovafloxacin against nosocomial gram-positive and gram-negative isolates. [*J. Antimicrob. Chemother.*](http://www.ncbi.nlm.nih.gov/pubmed/9222067), **1997**, *39*(Suppl. B), 29-34. |
| Olamufloxacin |   (S)-5-amino-7-(7-amino-5-azaspiro[2.4]heptan-5-yl)-1-cyclopropyl-6-fluoro-8-methyl-4-oxo-1,4-dihydroquinoline-3-carboxylic acid | [Takahashi, Y](http://www.ncbi.nlm.nih.gov/pubmed?term=Takahashi Y%5BAuthor%5D&cauthor=true&cauthor_uid=9174193).; [Masuda, N](http://www.ncbi.nlm.nih.gov/pubmed?term=Masuda N%5BAuthor%5D&cauthor=true&cauthor_uid=9174193).; [Otsuki, M](http://www.ncbi.nlm.nih.gov/pubmed?term=Otsuki M%5BAuthor%5D&cauthor=true&cauthor_uid=9174193).; [Miki, M](http://www.ncbi.nlm.nih.gov/pubmed?term=Miki M%5BAuthor%5D&cauthor=true&cauthor_uid=9174193).; [Nishino, T](http://www.ncbi.nlm.nih.gov/pubmed?term=Nishino T%5BAuthor%5D&cauthor=true&cauthor_uid=9174193). *In vitro* activity of HSR-903, a new quinolone. [*Antimicrob. Agents Chemother.*](http://www.ncbi.nlm.nih.gov/pubmed/9174193), **1997**,*41*(6), 1326-1330. |
| Pazufloxacin |   (S)-8-amino-10-(1-aminocyclopropyl)-9-fluoro-3-methyl-7-oxo-3,7-dihydro-2H-[1,4]oxazino[2,3,4-ij]quinoline-6-carboxylic acid | [Fukuoka, Y](http://www.ncbi.nlm.nih.gov/pubmed?term=Fukuoka Y%5BAuthor%5D&cauthor=true&cauthor_uid=8460909).; [Ikeda, Y](http://www.ncbi.nlm.nih.gov/pubmed?term=Ikeda Y%5BAuthor%5D&cauthor=true&cauthor_uid=8460909).; [Yamashiro, Y](http://www.ncbi.nlm.nih.gov/pubmed?term=Yamashiro Y%5BAuthor%5D&cauthor=true&cauthor_uid=8460909).; [Takahata, M](http://www.ncbi.nlm.nih.gov/pubmed?term=Takahata M%5BAuthor%5D&cauthor=true&cauthor_uid=8460909).; [Todo, Y](http://www.ncbi.nlm.nih.gov/pubmed?term=Todo Y%5BAuthor%5D&cauthor=true&cauthor_uid=8460909).; [Narita, H](http://www.ncbi.nlm.nih.gov/pubmed?term=Narita H%5BAuthor%5D&cauthor=true&cauthor_uid=8460909). *In vitro* and *in vivo* antibacterial activities of T-3761, a new quinolone derivative. [*Antimicrob. Agents Chemother.*](http://www.ncbi.nlm.nih.gov/pubmed/?term=Y.+Fukuoka+et+al.%2C+Antimicrob.+Ag.+Chemother.+37%2C+384+(1993).), **1993**, *37*(3), 384-392. |
| Pefloxacin |   1-ethyl-6-fluoro-7-(4-methylpiperazin-1-yl)-4-oxo-1,4-dihydroquinoline-3-carboxylic acid | [García-Rodríguez, J.A](http://www.ncbi.nlm.nih.gov/pubmed?term=García-Rodríguez JA%5BAuthor%5D&cauthor=true&cauthor_uid=2393288).; [García Sánchez, J.E](http://www.ncbi.nlm.nih.gov/pubmed?term=García Sánchez JE%5BAuthor%5D&cauthor=true&cauthor_uid=2393288).; [Muñoz Bellido, J.L](http://www.ncbi.nlm.nih.gov/pubmed?term=Muñoz Bellido JL%5BAuthor%5D&cauthor=true&cauthor_uid=2393288).; [Trujillano, I](http://www.ncbi.nlm.nih.gov/pubmed?term=Trujillano I%5BAuthor%5D&cauthor=true&cauthor_uid=2393288). *In vitro* activities of irloxacin and E-3846, two new quinolones. [*Antimicrob. Agents Chemother.*](http://www.ncbi.nlm.nih.gov/pubmed/2393288), **1990**, *34*(6), 1262-1267. |
| PGE-4175997 |   (R)-7-(3-(2-aminopropan-2-yl)pyrrolidin-1-yl)-1-cyclopropyl-8-methoxy-4-oxo-1,4-dihydroquinoline-3-carboxylic acid | [Barry, A.L](http://www.ncbi.nlm.nih.gov/pubmed?term=Barry AL%5BAuthor%5D&cauthor=true&cauthor_uid=11353655).; [Fuchs, P.C](http://www.ncbi.nlm.nih.gov/pubmed?term=Fuchs PC%5BAuthor%5D&cauthor=true&cauthor_uid=11353655).; [Brown, S.D](http://www.ncbi.nlm.nih.gov/pubmed?term=Brown SD%5BAuthor%5D&cauthor=true&cauthor_uid=11353655). *In vitro* activities of three nonfluorinated quinolones against representative bacterial isolates. [*Antimicrob. Agents Chemother.*](http://www.ncbi.nlm.nih.gov/pubmed/?term=Barry+AL%2C+Fuchs+PC%2C+Brown+SD.+Antimicrob.+Agents+Chemother.+2001%2C+45(6)%3A1923.), **2001**, *45*(6), 1923-1927. |
| PGE-9262932 |   7-((R)-3-((S)-1-aminoethyl)pyrrolidin-1-yl)-1-cyclopropyl-8-methoxy-4-oxo-1,4-dihydroquinoline-3-carboxylic acid | Same as for PGE-4175997 |
| PGE-9509924 |   (S)-7-(3-aminopiperidin-1-yl)-1-cyclopropyl-8-methoxy-4-oxo-1,4-dihydroquinoline-3-carboxylic acid | Same as for PGE-4175997 |
| Rufloxacin |   9-fluoro-10-(4-methylpiperazin-1-yl)-7-oxo-3,7-dihydro-2H-[1,4]thiazino[2,3,4-ij]quinoline-6-carboxylic acid | Same as for DW-116 |
| Sitafloxacin |   7-((S)-7-amino-5-azaspiro[2.4]heptan-5-yl)-8-chloro-6-fluoro-1-((1S,2R)-2-fluorocyclopropyl)-4-oxo-1,4-dihydroquinoline-3-carboxylic acid | Same as for DK-507k |
| Sparfloxacin |   5-amino-1-cyclopropyl-7-((3S,5R)-3,5-dimethylpiperazin-1-yl)-6,8-difluoro-4-oxo-1,4-dihydroquinoline-3-carboxylic acid | Same as for DW286 |
| Temafloxacin |   1-(2,4-difluorophenyl)-6-fluoro-7-(3-methylpiperazin-1-yl)-4-oxo-1,4-dihydroquinoline-3-carboxylic acid | Same as for enoxacin |
| Tosufloxacin |   7-(3-aminopyrrolidin-1-yl)-1-(2,4-difluorophenyl)-6-fluoro-4-oxo-1,4-dihydro-1,8-naphthyridine-3-carboxylic acid | Same as for DQ-113 |
| Trovafloxacin |   7-((1R,5S,6s)-6-amino-3-azabicyclo[3.1.0]hexan-3-yl)-1-(2,4-difluorophenyl)-6-fluoro-4-oxo-1,4-dihydro-1,8-naphthyridine-3-carboxylic acid | Same as for ABT-492 |
| Ulifloxacin |   6-fluoro-1-methyl-4-oxo-7-(piperazin-1-yl)-1,4-dihydro-[1,3]thiazeto[3,2-a]quinoline-3-carboxylic acid | [Ozaki, M](http://www.ncbi.nlm.nih.gov/pubmed?term=Ozaki M%5BAuthor%5D&cauthor=true&cauthor_uid=1667251).; [Matsuda, M](http://www.ncbi.nlm.nih.gov/pubmed?term=Matsuda M%5BAuthor%5D&cauthor=true&cauthor_uid=1667251).; [Tomii, Y](http://www.ncbi.nlm.nih.gov/pubmed?term=Tomii Y%5BAuthor%5D&cauthor=true&cauthor_uid=1667251).; [Kimura, K](http://www.ncbi.nlm.nih.gov/pubmed?term=Kimura K%5BAuthor%5D&cauthor=true&cauthor_uid=1667251).; [Kazuno, K](http://www.ncbi.nlm.nih.gov/pubmed?term=Kazuno K%5BAuthor%5D&cauthor=true&cauthor_uid=1667251).; [Kitano, M](http://www.ncbi.nlm.nih.gov/pubmed?term=Kitano M%5BAuthor%5D&cauthor=true&cauthor_uid=1667251).; [Kise, M](http://www.ncbi.nlm.nih.gov/pubmed?term=Kise M%5BAuthor%5D&cauthor=true&cauthor_uid=1667251).; [Shibata, K](http://www.ncbi.nlm.nih.gov/pubmed?term=Shibata K%5BAuthor%5D&cauthor=true&cauthor_uid=1667251).; [Otsuki, M](http://www.ncbi.nlm.nih.gov/pubmed?term=Otsuki M%5BAuthor%5D&cauthor=true&cauthor_uid=1667251).; [Nishino, T](http://www.ncbi.nlm.nih.gov/pubmed?term=Nishino T%5BAuthor%5D&cauthor=true&cauthor_uid=1667251).  *In vitro* antibacterial activity of a new quinolone, NM394. [*Antimicrob. Agents Chemother.*](http://www.ncbi.nlm.nih.gov/pubmed/1667251), **1991**, *35*(12), 2490-2495. |
| WIN57273 |   1-cyclopropyl-7-(2,6-dimethylpyridin-4-yl)-6-fluoro-4-oxo-1,4-dihydroquinoline-3-carboxylic acid | [Eliopoulos, G.M](http://www.ncbi.nlm.nih.gov/pubmed?term=Eliopoulos GM%5BAuthor%5D&cauthor=true&cauthor_uid=2393275).; [Klimm, K](http://www.ncbi.nlm.nih.gov/pubmed?term=Klimm K%5BAuthor%5D&cauthor=true&cauthor_uid=2393275).; [Rice, L.B](http://www.ncbi.nlm.nih.gov/pubmed?term=Rice LB%5BAuthor%5D&cauthor=true&cauthor_uid=2393275).; [Ferraro, M.J](http://www.ncbi.nlm.nih.gov/pubmed?term=Ferraro MJ%5BAuthor%5D&cauthor=true&cauthor_uid=2393275).; [Moellering, R.C. Jr](http://www.ncbi.nlm.nih.gov/pubmed?term=Moellering RC Jr%5BAuthor%5D&cauthor=true&cauthor_uid=2393275). Comparative *in vitro* activity of WIN 57273, a new fluoroquinolone antimicrobial agent. [*Antimicrob. Agents Chemother.*](http://www.ncbi.nlm.nih.gov/pubmed/2393275), **1990**, *34*(6), 1154-1159. |
