# Supplementary material for: Molecular Topology for the Discovery of New Broad-Spectrum Antibacterial Drugs
Source: Biomolecules. 2020 Sep 19;10(9):1343. doi: 10.3390/biom10091343 (PMC7564208; doi:10.3390/biom10091343)
Supplement: Supplementary file 1 [file biomolecules-10-01343-s001.zip › Table S2.doc]

**Table S2. Inactive group used in the topological pattern: Paper name/code, IUPAC name & structure and bibliograph**ic references about activity for each compound.

| **Paper name/code** | **IUPAC name & structure** | **Reference** |
| --- | --- | --- |
| Inact1 |   quinoline-8-carboxylic acid | Albert, A.; Rubbo, S.D.; Goldacre, J.R.; Balfour, B.G. The influence of chemical constitution on antibacterial activity. Part III: a study of 8-hydroxyquinoline (oxine) and related compounds. *Br. J. Exp. Pathol.*, **1947**, *28*(2), 69-87. |
| Benzoxiquine |   quinolin-8-yl benzoate | Khan, K.A.; Khan, S.A.; Khalid, S.M.; Ahmed, A.; Siddiqui, B.S.; Saleem, R.; Siddiqui, S.; Faizi, S. *In vitro* studies of the antibacterial and antifungal activities of oxine and its derivatives. *Arzneimittelforschung*., **1994**, *44*(8), 972-975. |
| Cinchophen |   2-phenylquinoline-4-carboxylic acid | [Strigácová, J](http://www.ncbi.nlm.nih.gov/pubmed?term=Strigácová J%5BAuthor%5D&cauthor=true&cauthor_uid=11347250).; [Hudecová, D](http://www.ncbi.nlm.nih.gov/pubmed?term=Hudecová D%5BAuthor%5D&cauthor=true&cauthor_uid=11347250).; [Varecka, L](http://www.ncbi.nlm.nih.gov/pubmed?term=Varecka L%5BAuthor%5D&cauthor=true&cauthor_uid=11347250).; [Lásiková, A](http://www.ncbi.nlm.nih.gov/pubmed?term=Lásiková A%5BAuthor%5D&cauthor=true&cauthor_uid=11347250).; [Végh, D](http://www.ncbi.nlm.nih.gov/pubmed?term=Végh D%5BAuthor%5D&cauthor=true&cauthor_uid=11347250). Some biological properties of new quinoline-4-carboxylic acid and quinoline-4-carboxamide derivatives. [*Folia Microbiol. (Praha)*](http://www.ncbi.nlm.nih.gov/pubmed/11347250), **2000**, *45*(4), 305-309.  Zimichev, A.V.; Zemtsova, M.N.; Kashaev, A.G.; Klimochkin, Y.N. Synthesis and antituberculous activity of quinoline isosteres of isoniazid. *Pharm. Chem. J.*, **2011**, *45*(4), 217-219. |
| Ferron |   8-hydroxy-7-iodoquinoline-5-sulfonic acid | Same as for Inact1 |
| Inact2 |   5-ethyl-6-hydroxy-8-oxo-5,8-dihydro-[1,3]dioxolo[4,5-g]quinoline-7-carboxylic acid | [Mitscher, L.A](http://www.ncbi.nlm.nih.gov/pubmed?term=Mitscher LA%5BAuthor%5D&cauthor=true&cauthor_uid=660597).; [Gracey, H.E](http://www.ncbi.nlm.nih.gov/pubmed?term=Gracey HE%5BAuthor%5D&cauthor=true&cauthor_uid=660597).; [Clark, G.W. 3rd](http://www.ncbi.nlm.nih.gov/pubmed?term=Clark GW 3rd%5BAuthor%5D&cauthor=true&cauthor_uid=660597); [Suzuki, T](http://www.ncbi.nlm.nih.gov/pubmed?term=Suzuki T%5BAuthor%5D&cauthor=true&cauthor_uid=660597). Quinolone antimicrobial agents. 1. Versatile new synthesis of 1-alkyl-1,4-dihydro-4-oxo-3-quinolinecarboxylic acids. [*J. Med. Chem.*](http://www.ncbi.nlm.nih.gov/pubmed/660597), **1978**, *21*(5), 485-489. |
| Inact3 |   5-ethyl-6-methyl-8-oxo-5,8-dihydro-[1,3]dioxolo[4,5-g]quinoline-7-carboxylic acid | Same as for Inact2 |
| Inact4 |   1-ethyl-6-methyl-4-oxo-1,2,3,4-tetrahydroquinoline-3-carboxylic acid | Same as for Inact2 |
| Inact5 |   1-ethyl-8-methyl-4-oxo-1,2,3,4-tetrahydroquinoline-3-carboxylic acid | Same as for Inact2 |
| Inact6 |   1-(3,4-bis(difluoro(hydroxy)methyl)phenyl)-6-fluoro-4-oxo-7-(piperazin-1-yl)-1,4-dihydroquinoline-3-carboxylic acid | [Xiao, W](http://www.ncbi.nlm.nih.gov/pubmed?term=Xiao W%5BAuthor%5D&cauthor=true&cauthor_uid=2778661).; [Krishnan, R](http://www.ncbi.nlm.nih.gov/pubmed?term=Krishnan R%5BAuthor%5D&cauthor=true&cauthor_uid=2778661).; [Lin, Y.I](http://www.ncbi.nlm.nih.gov/pubmed?term=Lin YI%5BAuthor%5D&cauthor=true&cauthor_uid=2778661).;  [Delos Santos, E.F](http://www.ncbi.nlm.nih.gov/pubmed?term=Delos Santos EF%5BAuthor%5D&cauthor=true&cauthor_uid=2778661).; [Kuck, N.A](http://www.ncbi.nlm.nih.gov/pubmed?term=Kuck NA%5BAuthor%5D&cauthor=true&cauthor_uid=2778661).;  [Babine, R.E](http://www.ncbi.nlm.nih.gov/pubmed?term=Babine RE%5BAuthor%5D&cauthor=true&cauthor_uid=2778661).; [Lang, S.A. Jr](http://www.ncbi.nlm.nih.gov/pubmed?term=Lang SA Jr%5BAuthor%5D&cauthor=true&cauthor_uid=2778661). Synthesis and *in vitro* antibacterial  activity of some 1-(difluoromethoxyphenyl)quinolone-3-carboxylic acids. *J. Pharm. Sci.*, **1989**, *78*(7), 585-588. |
| Inact7 |   1-(3,4-bis(difluoro(hydroxy)methyl)phenyl)-6-fluoro-7-(4-methylpiperazin-1-yl)-4-oxo-1,4-dihydroquinoline-3-carboxylic acid | Same as for Inact6 |
| Inact8 |   1-ethyl-6-fluoro-7-(3-methylthiazolidin-4-yl)-4-oxo-1,4-dihydroquinoline-3-carboxylic acid | Zhang, M.; Haemers, A.; Vanden Berghe, D.; Pattyn, S.; Bollaert, W.; Levshin, I. [Quinolone antibacterials. 1. 7-(2-substituted-4-thiazolyl- and thiazolidinyl)quinolones.](https://scifinder.cas.org/scifinder/references/answers/18BC26A6X86F350B0X5AF211B7425C38B6E4:190B39ACX86F350B0X48F0144C1B7AAD8572/1.html?nav=eNpb85aBtYSBMbGEQcXQ0sDJ2NLROcLCzM3Y1MDJIMLEws3A0MTE2dDJ3NHRxcLU3AioNKm4iEEwK7EsUS8nMS9dzzOvJDU9tUjo0YIl3xvbLZgYGD0ZWMsSc0pTK4oYBBDq_Epzk1KL2tZMleWe8qCbiYGhooCBgSEYaGBGCYO0Y2iIh39QvKdfmKtfCJDh5x_vHuQfGuDp517CwJmZW5BfVAI0obiQoY6BGaiPAagvH85jLGFgLkoqQnWYU35-Tmpi3lmFooarc369AzosCuawAqB2pqJ8kHY-IKu4GKhTO78oXS85sVgvvzg5sUivOLWoLLVILyU_NzEzTy85Pzc3P08vGOiK4ILUZJsJqxfITn9wiomByYeBJ7fSvyglMy8xxzu1soRBwwdokD7QIH2wQfoQg_QhBulDDNIHqrT2YWDPrQSZWFzCIOkDcrd-aUlmjr5PZl52aopHYnFGcGqJdUVBAdBx4mBvgaT1UKRv5FyPmnpXSw4UnDDPg1VB5Xe5tE6dtmO7CTMouMt5gEElYO_AAAYVwDArLk4EhwEAJaul5A&key=caplus_1991:471455&title=UXVpbm9sb25lIGFudGliYWN0ZXJpYWxzLiAgMS4gIDctKDItU3Vic3RpdHV0ZWQtNC10aGlhem9seWwtIGFuZCAtdGhpYXpvbGlkaW55bClxdWlub2xvbmVz&launchSrc=reflist&p=1) *J. Heterocyclic Chem.*, **1991**, *22*(38), 673-683. |
| Inact9 |   7-(2,3-dimethylthiazolidin-4-yl)-1-ethyl-6-fluoro-4-oxo-1,4-dihydroquinoline-3-carboxylic acid | Same as for Inact8 |
| Inact10 |   7-ethyl-3-methyl-4-oxo-4,7-dihydroisothiazolo[5,4-b]pyridine-5-carboxylic acid | Richardson, T.O.; Neale, N.; Carwell, N. Synthesis and antibacterial activity of 7-ethyl-3-methyl-4,7-dihydro-4-oxoisothiazolo[5,4-b]pyridine-5-carboxylic acid. *J. Heterocyclic Chem.*, **1995**, *32*(1), 359-361. |
| Inact11 |   7-amino-4-(4-methoxyphenyl)-5-methyl-6-(4-methylpiperazin-1-yl)-1-oxo-1,4-dihydronaphthalene-2-carboxylic acid | [Cecchetti, V](http://www.ncbi.nlm.nih.gov/pubmed?term=Cecchetti V%5BAuthor%5D&cauthor=true&cauthor_uid=8558512).; [Fravolini, A](http://www.ncbi.nlm.nih.gov/pubmed?term=Fravolini A%5BAuthor%5D&cauthor=true&cauthor_uid=8558512).; [Lorenzini, M.C](http://www.ncbi.nlm.nih.gov/pubmed?term=Lorenzini MC%5BAuthor%5D&cauthor=true&cauthor_uid=8558512).; [Tabarrini, O](http://www.ncbi.nlm.nih.gov/pubmed?term=Tabarrini O%5BAuthor%5D&cauthor=true&cauthor_uid=8558512).; [Terni, P](http://www.ncbi.nlm.nih.gov/pubmed?term=Terni P%5BAuthor%5D&cauthor=true&cauthor_uid=8558512).; [Xin, T](http://www.ncbi.nlm.nih.gov/pubmed?term=Xin T%5BAuthor%5D&cauthor=true&cauthor_uid=8558512). Studies on 6-aminoquinolones: synthesis and antibacterial evaluation of 6-amino-8-methylquinolones. [*J. Med. Chem.*](http://www.ncbi.nlm.nih.gov/pubmed/8558512), **1996**, *39*(2), 436-445. |
| Inact12 |   7-amino-4-cyclopropyl-5-ethyl-6-(4-methylpiperazin-1-yl)-1-oxo-1,4-dihydronaphthalene-2-carboxylic acid | [Cecchetti, V](http://www.ncbi.nlm.nih.gov/pubmed?term=Cecchetti V%5BAuthor%5D&cauthor=true&cauthor_uid=10632056).; [Tabarrini, O](http://www.ncbi.nlm.nih.gov/pubmed?term=Tabarrini O%5BAuthor%5D&cauthor=true&cauthor_uid=10632056).; [Sabatini, S](http://www.ncbi.nlm.nih.gov/pubmed?term=Sabatini S%5BAuthor%5D&cauthor=true&cauthor_uid=10632056).; [Miao, H](http://www.ncbi.nlm.nih.gov/pubmed?term=Miao H%5BAuthor%5D&cauthor=true&cauthor_uid=10632056).; [Filipponi, E](http://www.ncbi.nlm.nih.gov/pubmed?term=Filipponi E%5BAuthor%5D&cauthor=true&cauthor_uid=10632056).; [Fravolini, A](http://www.ncbi.nlm.nih.gov/pubmed?term=Fravolini A%5BAuthor%5D&cauthor=true&cauthor_uid=10632056). Studies on 6-aminoquinolones: synthesis and antibacterial evaluation of 6-amino-8-ethyl- and 6-amino-8-methoxyquinolones. [*Bioorg. Med. Chem.*](http://www.ncbi.nlm.nih.gov/pubmed/10632056), **1999**, *7*(11), 2465-2471. |
| Inact13 |   1-cyclopropyl-6-fluoro-7-(4-(5-(2-nitrophenyl)-1,3,4-thiadiazol-2-yl)piperazin-1-yl)-4-oxo-1,2,3,4-tetrahydroquinoline-3-carboxylic acid | [Foroumadi, A](http://www.ncbi.nlm.nih.gov/pubmed?term=Foroumadi A%5BAuthor%5D&cauthor=true&cauthor_uid=14505733).; [Soltani, F](http://www.ncbi.nlm.nih.gov/pubmed?term=Soltani F%5BAuthor%5D&cauthor=true&cauthor_uid=14505733).; [Moshafi, M.H](http://www.ncbi.nlm.nih.gov/pubmed?term=Moshafi MH%5BAuthor%5D&cauthor=true&cauthor_uid=14505733).; [Ashraf-Askari, R](http://www.ncbi.nlm.nih.gov/pubmed?term=Ashraf-Askari R%5BAuthor%5D&cauthor=true&cauthor_uid=14505733). Synthesis and *in vitro* antibacterial activity of some N-(5-aryl-1,3,4-thiadiazole-2-yl)piperazinyl quinolone derivatives. *Farmaco*, **2003**, *58* (10), 1023-1028. |
| Inact14 |   1-ethyl-6-fluoro-7-(4-(5-(2-nitrophenyl)-1,3,4-thiadiazol-2-yl)piperazin-1-yl)-4-oxo-1,2,3,4-tetrahydroquinoline-3-carboxylic acid | Same as for Inact13 |
| Inact15 |   1-ethyl-6-fluoro-7-(4-(5-(2-nitrophenyl)-1,3,4-thiadiazol-2-yl)piperazin-1-yl)-4-oxo-1,2,3,4-tetrahydro-1,8-naphthyridine-3-carboxylic acid | Same as for Inact13 |
| Inact16 |   1-cyclopropyl-6-fluoro-7-(4-(5-(3-nitrophenyl)-1,3,4-thiadiazol-2-yl)piperazin-1-yl)-4-oxo-1,2,3,4-tetrahydroquinoline-3-carboxylic acid | Same as for Inact13 |
| Inact17 |   1-ethyl-6-fluoro-7-(4-(5-(3-nitrophenyl)-1,3,4-thiadiazol-2-yl)piperazin-1-yl)-4-oxo-1,2,3,4-tetrahydroquinoline-3-carboxylic acid | Same as for Inact13 |
| Inact18 |   1-ethyl-6-fluoro-7-(4-(5-(3-nitrophenyl)-1,3,4-thiadiazol-2-yl)piperazin-1-yl)-4-oxo-1,2,3,4-tetrahydro-1,8-naphthyridine-3-carboxylic acid | Same as for Inact13 |
| Inact19 |   1-cyclopropyl-6-fluoro-7-(4-(5-(4-nitrophenyl)-1,3,4-thiadiazol-2-yl)piperazin-1-yl)-4-oxo-1,2,3,4-tetrahydroquinoline-3-carboxylic acid | Same as for Inact13 |
| Inact20 |   1-ethyl-6-fluoro-7-(4-(5-(4-nitrophenyl)-1,3,4-thiadiazol-2-yl)piperazin-1-yl)-4-oxo-1,2,3,4-tetrahydroquinoline-3-carboxylic acid | Same as for Inact13 |
| Inact21 |   1-ethyl-6-fluoro-7-(4-(5-(4-nitrophenyl)-1,3,4-thiadiazol-2-yl)piperazin-1-yl)-4-oxo-1,2,3,4-tetrahydro-1,8-naphthyridine-3-carboxylic acid | Same as for Inact13 |
| Inact22 |   1-ethyl-6-fluoro-7-(4-(5-((2-nitrobenzyl)sulfonyl)-1,3,4-thiadiazol-2-yl)piperazin-1-yl)-4-oxo-1,4-dihydroquinoline-3-carboxylic acid | [Foroumadi, A](http://www.ncbi.nlm.nih.gov/pubmed?term=Foroumadi A%5BAuthor%5D&cauthor=true&cauthor_uid=16105736).; [Emami, S](http://www.ncbi.nlm.nih.gov/pubmed?term=Emami S%5BAuthor%5D&cauthor=true&cauthor_uid=16105736).; [Hassanzadeh, A](http://www.ncbi.nlm.nih.gov/pubmed?term=Hassanzadeh A%5BAuthor%5D&cauthor=true&cauthor_uid=16105736).; [Rajaee, M](http://www.ncbi.nlm.nih.gov/pubmed?term=Rajaee M%5BAuthor%5D&cauthor=true&cauthor_uid=16105736).; [Sokhanvar, K](http://www.ncbi.nlm.nih.gov/pubmed?term=Sokhanvar K%5BAuthor%5D&cauthor=true&cauthor_uid=16105736).; [Moshafi, M.H](http://www.ncbi.nlm.nih.gov/pubmed?term=Moshafi MH%5BAuthor%5D&cauthor=true&cauthor_uid=16105736).; [Shafiee, A](http://www.ncbi.nlm.nih.gov/pubmed?term=Shafiee A%5BAuthor%5D&cauthor=true&cauthor_uid=16105736). Synthesis and antibacterial activity of  N-(5-benzylthio-1,3,4-thiadiazol-2-yl)  and N-(5-benzylsulfonyl-1,3,4-  thiadiazol-2-yl)piperazinyl quinolone derivatives. [*Bioorg. Med. Chem. Lett.*](http://www.ncbi.nlm.nih.gov/pubmed/16105736), **2005**, *15*(20), 4488-4492. |
| Inact23 |   1-ethyl-6-fluoro-7-(4-(5-((3-nitrobenzyl)sulfonyl)-1,3,4-thiadiazol-2-yl)piperazin-1-yl)-4-oxo-1,4-dihydroquinoline-3-carboxylic acid | Same as for Inact22 |
| KB-5246 |   6-fluoro-5-(4-methylpiperazin-1-yl)-4-oxa-1-thia-2a1-azacyclopenta[cd]phenalen-8(3H)-one | Taguchi, M.; Kondo, H.; Inoue, Y.; Kawahata, Y.; Jinbo, Y.; Sakamoto, F.; Tsukamoto, G.  Synthesis and antibacterial activity of new tetracyclic quinolone antibacterials. *J. Med. Chem.*, **1992**, *35*(1), 94-99. |
| Inact25 |   6-fluoro-5-(4-methylpiperazin-1-yl)-8-oxo-3,8-dihydro-4-oxa-1-thia-2a1-azacyclopenta[cd]phenalene-9-sulfonic acid | Same as for Inact24 |
| Inact26 |   1-(4-(4-chlorophenyl)-3-(1H-pyrrol-1-yl)-5,6,7,8-tetrahydrothieno[2,3-b]quinolin-2-yl)ethanone | Geies, A.A.; Bakhite, E.A.; el-Kashef, H.S. Synthesis and antimicrobial activities of some new pyrrolylthieno[2,3-b]-quinoline derivatives. *Pharmazie*, **1998**, *53*(10), 686-690. |
| Inact27 |   4-(4-chlorophenyl)-2-(1,5-diphenyl-4,5-dihydro-1H-pyrazol-3-yl)-3-(1H-pyrrol-1-yl)-5,6,7,8-tetrahydrothieno[2,3-b]quinoline | Same as for Inact26 |
| Inact28 |   6-(4-(4-chlorophenyl)-3-(1H-pyrrol-1-yl)-5,6,7,8-tetrahydrothieno[2,3-b]quinolin-2-yl)-4-phenyl-3,4-dihydropyrimidine-2(1H)-thione | Same as for Inact26 |
| Inact29 |   4-(4-chlorophenyl)-2-(5-(methylthio)-4-phenyl-4H-1,2,4-triazol-3-yl)-3-(1H-pyrrol-1-yl)-5,6,7,8-tetrahydrothieno[2,3-b]quinoline | Same as for Inact26 |
| Inact30 |   6-fluoro-2-phenyl-7-(piperidin-1-yl)-4H-chromen-4-one | Mari, S.; Rossi, M.; Valenti, P.; Da Re, P. Flavone and xanthone derivatives related to fluoroquinolones. *Farmaco*, **1999**, *54*(6), 411–415. |
| Inact31 |   6-fluoro-2-phenyl-7-(piperazin-1-yl)-4H-chromen-4-one | Same as for Inact30 |
| Inact32 |   6-fluoro-7-(4-methylpiperazin-1-yl)-2-phenyl-4H-chromen-4-one | Same as for Inact30 |
| Inact33 |   6-fluoro-7-(4-(2-hydroxyethyl)piperazin-1-yl)-2-phenyl-4H-chromen-4-one | Same as for Inact30 |
| Inact34 |   6-fluoro-7-(4-(2-methoxyphenyl)piperazin-1-yl)-2-phenyl-4H-chromen-4-one | Same as for Inact30 |
| Inact35 |   7-amino-10-ethyl-1-hydroxy-3-methoxy-6-(4-methylpiperazin-1-yl)acridin-9(10H)-one | [Tabarrini, O](http://www.ncbi.nlm.nih.gov/pubmed?term=Tabarrini O%5BAuthor%5D&cauthor=true&cauthor_uid=10377219).; [Cecchetti, V](http://www.ncbi.nlm.nih.gov/pubmed?term=Cecchetti V%5BAuthor%5D&cauthor=true&cauthor_uid=10377219).; [Fravolini, A](http://www.ncbi.nlm.nih.gov/pubmed?term=Fravolini A%5BAuthor%5D&cauthor=true&cauthor_uid=10377219).; [Nocentini, G](http://www.ncbi.nlm.nih.gov/pubmed?term=Nocentini G%5BAuthor%5D&cauthor=true&cauthor_uid=10377219).; [Barzi, A](http://www.ncbi.nlm.nih.gov/pubmed?term=Barzi A%5BAuthor%5D&cauthor=true&cauthor_uid=10377219).; [Sabatini, S](http://www.ncbi.nlm.nih.gov/pubmed?term=Sabatini S%5BAuthor%5D&cauthor=true&cauthor_uid=10377219).; [Miao, H](http://www.ncbi.nlm.nih.gov/pubmed?term=Miao H%5BAuthor%5D&cauthor=true&cauthor_uid=10377219).; [Sissi, C](http://www.ncbi.nlm.nih.gov/pubmed?term=Sissi C%5BAuthor%5D&cauthor=true&cauthor_uid=10377219). Design and synthesis of modified quinolones as antitumoral acridones. [*J. Med. Chem.*](http://www.ncbi.nlm.nih.gov/pubmed/10377219), **1999**, *42*(12), 2136-2144. |
| Inact36 |   7-amino-10-ethyl-1,3-dihydroxy-6-(4-methylpiperazin-1-yl)acridin-9(10H)-one | Same as for Inact35 |
| Inact37 |   7-amino-10-ethyl-1,3-dihydroxy-6-(pyrrolidin-1-yl)acridin-9(10H)-one | Same as for Inact35 |
| Inact38 |   7-amino-10-ethyl-1,3-dihydroxy-6-thiomorpholinoacridin-9(10H)-one | Same as for Inact35 |
| Inact39 |   7-amino-10-(2-(diethylamino)ethyl)-1,3-dihydroxy-6-(4-methylpiperazin-1-yl)acridin-9(10H)-one | Same as for Inact35 |
| Inact40 |   7-amino-6-chloro-10-ethyl-1,3-dihydroxyacridin-9(10H)-one | Same as for Inact35 |
| Inact41 |   1-benzyl-6-fluoro-3-hydroxy-7-(pyrrolidin-1-yl)quinazoline-2,4(1H,3H)-dione | [Tran, T.P](http://www.ncbi.nlm.nih.gov/pubmed?term=Tran TP%5BAuthor%5D&cauthor=true&cauthor_uid=15357962).; [Ellsworth, E.L](http://www.ncbi.nlm.nih.gov/pubmed?term=Ellsworth EL%5BAuthor%5D&cauthor=true&cauthor_uid=15357962).; [Stier, M.A](http://www.ncbi.nlm.nih.gov/pubmed?term=Stier MA%5BAuthor%5D&cauthor=true&cauthor_uid=15357962).; [Domagala, J.M](http://www.ncbi.nlm.nih.gov/pubmed?term=Domagala JM%5BAuthor%5D&cauthor=true&cauthor_uid=15357962).; [Showalter, H.D](http://www.ncbi.nlm.nih.gov/pubmed?term=Hollis Showalter HD%5BAuthor%5D&cauthor=true&cauthor_uid=15357962).; [Gracheck, S.J](http://www.ncbi.nlm.nih.gov/pubmed?term=Gracheck SJ%5BAuthor%5D&cauthor=true&cauthor_uid=15357962).; [Shapiro, M.A](http://www.ncbi.nlm.nih.gov/pubmed?term=Shapiro MA%5BAuthor%5D&cauthor=true&cauthor_uid=15357962).; [Joannides, T.E](http://www.ncbi.nlm.nih.gov/pubmed?term=Joannides TE%5BAuthor%5D&cauthor=true&cauthor_uid=15357962).; [Singh, R](http://www.ncbi.nlm.nih.gov/pubmed?term=Singh R%5BAuthor%5D&cauthor=true&cauthor_uid=15357962). Synthesis and structural-activity relationships of 3-hydroxyquinazoline-2,4-dione antibacterial agents. [*Bioorg. Med. Chem. Lett.*](http://www.ncbi.nlm.nih.gov/pubmed/15357962), **2004**, *14*(17), 4405-4409. |
| Inact42 |   1-(2,4-difluorophenyl)-6-fluoro-3-hydroxy-7-(pyrrolidin-1-yl)quinazoline-2,4(1H,3H)-dione | Same as for Inact41 |
| Naptalam |   2-(naphthalen-1-ylcarbamoyl)benzoic acid | Shahid, K.; Ali, S.; Shahzadi, S. The chemistry, properties, and characterization of organotin(IV) complexes of 2-(N-naphthylamido)benzoic acid. *J. Coord. Chem.* **2009**, *62*(17), 2919-2926. |
| PGE-5215205 |   1-methyl-4-oxo-1,4-dihydroquinoline-3-carboxylic acid | [Macinga, D.R](http://www.ncbi.nlm.nih.gov/pubmed?term=Macinga DR%5BAuthor%5D&cauthor=true&cauthor_uid=12878515).; [Renick, P.J](http://www.ncbi.nlm.nih.gov/pubmed?term=Renick PJ%5BAuthor%5D&cauthor=true&cauthor_uid=12878515).; [Makin, K.M](http://www.ncbi.nlm.nih.gov/pubmed?term=Makin KM%5BAuthor%5D&cauthor=true&cauthor_uid=12878515).; [Ellis, D.H](http://www.ncbi.nlm.nih.gov/pubmed?term=Ellis DH%5BAuthor%5D&cauthor=true&cauthor_uid=12878515).; [Kreiner, A.A](http://www.ncbi.nlm.nih.gov/pubmed?term=Kreiner AA%5BAuthor%5D&cauthor=true&cauthor_uid=12878515).; [Li, M](http://www.ncbi.nlm.nih.gov/pubmed?term=Li M%5BAuthor%5D&cauthor=true&cauthor_uid=12878515).; [Rupnik, K.J](http://www.ncbi.nlm.nih.gov/pubmed?term=Rupnik KJ%5BAuthor%5D&cauthor=true&cauthor_uid=12878515).; [Kincaid, E.M](http://www.ncbi.nlm.nih.gov/pubmed?term=Kincaid EM%5BAuthor%5D&cauthor=true&cauthor_uid=12878515).; [Wallace, C.D](http://www.ncbi.nlm.nih.gov/pubmed?term=Wallace CD%5BAuthor%5D&cauthor=true&cauthor_uid=12878515).; [Ledoussal, B](http://www.ncbi.nlm.nih.gov/pubmed?term=Ledoussal B%5BAuthor%5D&cauthor=true&cauthor_uid=12878515).; [Morris, T.W](http://www.ncbi.nlm.nih.gov/pubmed?term=Morris TW%5BAuthor%5D&cauthor=true&cauthor_uid=12878515). Unique biological properties and molecular mechanism of 5,6-bridged quinolones. *Antimicrob. Agents Chemother.*, **2003**, *47*(8), 2526-2537. |
| PGE-6116542 |   7,8-dimethyl-10-oxo-7,10-dihydro-1H-[1,3]dioxino[5,4-f]quinoline-9-carboxylic acid | Same as for PGE-5215205 |
| Quinoline |   quinoline | Darawsheh, M.; Abu Ali, H.; Abuhijleh, A.L.; Rappocciolo, E.; Akkawi, M.; Jaber, S.; Maloul, S.; Hussein, Y. New mixed ligand zinc (II) complexes based on the antiepileptic drug sodium valproate and bioactive nitrogen-donor ligands. Synthesis, structure and biological properties. *Eur. J. Med. Chem.*, **2014**, *82*, 152-163. |
