# Supplementary material for: Molecular Topology for the Discovery of New Broad-Spectrum Antibacterial Drugs
Source: Biomolecules. 2020 Sep 19;10(9):1343. doi: 10.3390/biom10091343 (PMC7564208; doi:10.3390/biom10091343)
Supplement: Supplementary file 1 [file biomolecules-10-01343-s001.zip › Table S3.doc]

**^Table S3. Symbols and Definitions of Topological Indices used with DESMOL13a and MOLCONN-Zb programs.^**

| **Symbol** | **Name** | **Definition** | **Ref.** |
| --- | --- | --- | --- |
| ***Connectivity Indices*** | | | |
| *^k^*χ*_t_*  *k*=0-10  *t*=p,c,pc | Kier-Hall indices of order *k* and type path (p), cluster (c) and path-cluster (pc) | ${}^{k}\chi_{t}=\sum_{j=1}^{{}^{k}n_{t}} \left(\prod_{i\in\text{S}_{j}} \delta_{i}\right)^{-1/2}$ *δ_i_*: number of bonds, *σ* o *π*, of the atom *i* to non-hydrogen atoms.  S*_j_*_,_: *j*th substructure of order *k* and type *t*. | c |
| *^k^*χ*_t_*^v^  *k*=0-10  *t*=p,c,pc | Kier-Hall indices of order *k* and type path (p), cluster (c) and path-cluster (pc) | ${}^{k}\chi_{t}^{\text{v}}=\sum_{j=1}^{{}^{k}n_{t}} \left(\prod_{i\in\text{S}_{j}} \delta_{i}^{\text{v}}\right)^{-1/2}$ *δ_i_*^v^: Kier-Hall valence of the atom *i*.  S*_j_*_,_: *j*th substructure of order *k* and type *t*. | b |
| ^k^D_t_  k=0-10  t=p,c,pc | Connectivity differences of order *k* and type path (p), cluster (c) and path-cluster (pc) | $^{k}\text{D}_{t}={}^{k}\chi_{t}-{}^{k}\chi_{t}^{\text{v}}$ | b |
| ^k^C_t_  k=0-10  t=p,c,pc | Connectivity ratios of order *k* and type path (p), cluster (c) and path-cluster (pc) | $^{k}\text{C}_{t}={}^{k}\chi_{t}/{}^{k}\chi_{t}^{\text{v}}$ | b |
| ***Charge Indices*** | | | |
| G*_k_*  *k*=1-5 | Topological charge ndices of order *k* | $G_{k}=\sum_{i=1}^{N-1} \sum_{j=i+1}^{N} \left\vert\mathbf{M}_{ij}-\mathbf{M}_{ji}\right\vert\delta(k,\mathbf{D}_{ij})$ **M**=**A.Q**: product of the adjacency and inverse squared distance matrices for the hydrogen-depleted molecular graph.  **D**: distance matrix.  *δ*: Kronecker delta. | d |
| G*_k_*^v^  *k*=1-5 | Valence topological charge ndices of order *k* | $G_{k}^{\text{v}}=\sum_{i=1}^{N-1} \sum_{j=i+1}^{N} \left\vert\mathbf{M}_{ij}^{\text{v}}-\mathbf{M}_{ji}^{\text{v}}\right\vert\delta(k,\mathbf{D}_{ij})$ **M**^v^=**A^v^.Q**: product of the electronegativity-modified adjacency and inverse squared distance matrices for the hydrogen-depleted molecular graph.  **D**: distance matrix.  *δ*: Kronecker delta. | d |
| J*_k_*, J*_k_*^v^  *k*=1-5 | Pondered topological charge ndices of order *k* | $\text{J}_{k}=\frac{\text{G}_{k}}{\text{N}-1}$ $\text{J}_{k}^{\text{v}}=\frac{\text{G}_{k}^{\text{v}}}{\text{N}-1}$ | d |
| ***Electrotopological Indices*** | | | |
| S(i) | Sum of electrotopological indices for a type of atom *i* | $S_{i}=I_{i}+\Delta I_{i}$ I_i_: intrinsic state value of atom *i*.  ΔI_i_: perturbation of *I_j_* on *I_i_* with the form as *ΔI_ij_ = (I_i_-I_j_)/D^2^_ij_* | e |
| Gmaxpos | Gmaxpos index | Maximum positive value for the electrotopological state of non-hydrogen atoms in the molecule. | f |
| Hmaxpos | Hmaxpos index | Maximum positive value for the electrotopological state of hydrogen atoms in the molecule. | c |
| ***Molecular Properties*** | | | |
| N | Molecular size | Number of non-hydrogen atoms. | g |
| L | Length | Maximum distance between atoms in terms of bonds. | h |
| PR_i_ | PR0 a PR3 | Number of pairs of ramifications separated by *i* atoms. | f |
| R | Ramification | Number of simple structural branches. | f |
| V*_k_*  *k*=3,4 | Vertexes of grade *k* | Number of atoms with *k* bonds, *σ* o *π*, with other atoms (hydrogens not incluided). | f |
| knotp | knotp index | Difference between *^3^*χ*_c_* and *^4^*χ*_pc_* indices | i |
| knotpv | knotpv index | Difference between *^3^*χ^v^*_c_* y *^4^*χ^v^*_pc_* indices | h |
| numhbd | numhbd index | Number of hydrogen-donating atoms in the molecule. | h |
| numhba | numhbd index | Number of hydrogen-accepting atoms in the molecule. | h |
| ***Information Indices*** | | | |
| I_Shannon_ | Shannon index | Índex based on the atomic diversity of the molecule. | g |
| NI, NI2 | NI, NI2 indices | Indices based on information theory. | g |
| BonIdW | Índice Bonchev IdW(G) | Index based in the distribution of topological distances in the molecule. | j |
| BonIdG | Índice Bonchev Id(G) | Index based on the number of order 2 subgraphs in the molecule. | i |
| ***Molecular Shape Indices*** | | | |
| *^n^*κ  *n*=1-3 | Kappa index of order *n* | ^1^κ = N(N-1)^2^/(^1^P)^2^  ^2^κ = (N-1)(N-2)^2^/(^2^P)^2^  ^3^κ = (N-3)(N-2)^2^/(^3^P)^2^ [N even; N>3]  ^3^κ = (N-1)(N-3)^2^/(^3^P)^2^ [N odd; N>3]  N: number of non-hydrogen atoms.  ^1,2,3^P : number of type path subgraphs of order 1,2,3. | k |
| *^n^*κ_α_  *n*=1-3 | Kappa-alpha index of order *n* | ^1^κ = N+α(N+α-1)^2^/(^1^P+α)^2^  ^2^κ = (N+α-1)(N+α-2)^2^/(^2^P+α)^2^  ^3^κ = (N+α-3)(N+α-2)^2^/(^3^P+α)^2^ [N pares; N>3]  ^3^κ = (N+α-1)(N+α-3)^2^/(^3^P+α)^2^ [N impares; N>3]  N: number of non-hydrogen atoms.  ^1,2,3^P : number of type path subgraphs of order 1,2,3.  α = Σ[(*R_i_ / R_Csp3_* )-1]  R_i_: covalent radius for atom *i*.  R_Csp3_: covalent radius for atom *Csp^3^*. | l |
| Φ | Phia flexibility index | Φ = (^1^κ_α_ ^. 2^κ_α_) / N  N: number of non-hydrogen atoms. | m |
| ***Global Topological Indices*** | | | |
| Sum-I | Sum of the intrinsic state values | $I_{i}=\frac{\delta_{i}^{v}+1}{\delta_{i}}$ ; $Sum-I=\sum I_{i}$ *δ_i_*, number of bonds, *σ* o *π*, of the atom *i* to non-hydrogen atoms.  *δ_i_*^v^, Kier-Hall valence of the atom *i*. | e |
| Sum-ΔI | Sum of the change in intrinsic state values | $\Delta I=\frac{I_{i}-I_{j}}{r_{ij}^{2}}$ ; $Sum-\Delta I=\sum\frac{I_{i}-I_{j}}{r_{ij}^{2}}$ r_ij_, number of vertexes between *i* and *j* atoms. | e |
| TETS2 | Total electrotopological state index | Sum of all the electrotopological indices in the molecule. | e |
| TTd4 | TTd4 index | Sum of the intrinsic state of all the atoms in the molecule. | e |
| nclass | Nclass index | Maximum number of topological vertices of a graph by the shortest path | n |
| W | Wiener index | Sum of the topological distances between all the non-hydrogen atoms by the shortest path. | o |
| Wp | Wiener polarity index | Number of pairs of atoms with a distance of 3 bonds. | p |
| Ww | Hyper-Wiener index | Sum of the topological distances and the squared topological distances between all the non-hydrogen atoms by the shortest path. | q |
| Wt | Total Wiener index | Sum of the topological distances between all the non-hydrogen atoms by the longest path. | r |
| PlattF | PlattF index | Sum of the grades of the bonds. | s |

a. DESMOL13 software; Unidad de Investigación de Diseño de Fármacos y Conectividad Molecular, Facultad de Farmacia, Universitat de València: Valencia, **2000**. Ratios and differences connectivity indices were calculated manually using the Microsoft Excel program.

b. Hall, L. H. MOLCONN-Z software; Eastern Nazarene College: Quincy (Massachusetts), **1995**.

c. [Kier, L.B](http://www.ncbi.nlm.nih.gov/pubmed?term=Kier LB%5BAuthor%5D&cauthor=true&cauthor_uid=6644566).; [Hall, L.H](http://www.ncbi.nlm.nih.gov/pubmed?term=Hall LH%5BAuthor%5D&cauthor=true&cauthor_uid=6644566). General definition of valence delta-values for molecular connectivity. *[J. Pharm.](http://www.ncbi.nlm.nih.gov/pubmed/6644566#%23) Sci.*, **1983**, *72*(10), 1170-1173.

d. Gálvez, J.; García-Domenech, R.; Salabert, M.T.; Soler, R. Charge indexes. New topological descriptors. *J. Chem. Inf. Comput. Sci.*, **1994**, *34(3)*, 520-525.

e. Kier, L.B.; Hall, L.H. The E-state as an extended free valence. *J. Chem. Inf. Comput. Sci.*, **1997**, *37*(3), 548-552.

f. Basak, S.C.; Mills, D. Quantitative structure-property relationships (QSPRs) for the estimation of vapor pressure: a hierarchical approach using mathematical structural descriptors. *J. Chem. Inf. Comput. Sci.*, **2001**, *41*(3), 692-701.

g. Shannon, C.E.; Weaver, W. *The Mathematical Theory of Communication*; University of Illinois Press: Urbana, **1949**.

h. Moliner, R.; Garcia, F.; Galvez, J.; Garcia-Domenech, R.; Serrano, C. Nuevos índices topológicos en conectividad molecular. Su aplicación a algunas propiedades fisicoquímicas de un grupo de hidrocarburos alifáticos. *An. Real Acad. Farm.*, **1991**, *57*, 287-298.

i. Cummins, D.J.; Andrews, C.W.; Bentley, J.A.; Cory, M. Molecular diversity in chemical databases: comparison of medicinal chemistry knowledge bases and databases of commercially available compounds. *J. Chem. Inf. Comput. Sci.*, **1996**, *36*(4), 750-763.

j. Bonchev, D.; Trinajstic, N. On topological characterization of molecular branching. *Int. J. Quantum Chem.*, **1978**, *14*(S12), 293-303.

k. Kier, L.B. A shape [index from molecular graphs](http://scholar.google.es/scholar?cluster=14129905811510536866&hl=es&as_sdt=0&sciodt=0). *Quant. Struct.-Act. Relat.*, **1985**, *4*(3), 109-116.

l. Kier, L.B. Distinguishing atom differences in a molecular graph shape index. *Quant. Struct.-Act. Relat.*, **1986**, *5*(1), 7-12.

m. Kier, L.B. An index of molecular flexibility from Kappa shape attributes. *Quant. Struct.-Act. Relat.*, **1989**, *8*(3), 221-224.

n. Suay-García, B.; Alemán-López, P.; Bueso-Bordils, J.I.; Falcó, A.; Pérez-Gracia, M.T.; Antón-Fos, G.M. opological index Nclass as a factor determining the antibacterial activity of quinolones against *Escherichia coli*. *Future Med. Chem.* **2019**, *11*(17), 2255-2262.

o. Wiener, H. Structural determination of paraffin boiling points. *J. Am. Chem. Soc.*, **1947**, *69*(1), 17-20.

p. Wiener, H. Relation of the physical properties of the isomeric alkanes to molecular structure. Surface tension, specific dispersion, and critical solution temperature in aniline. *J. Phys. Chem.*, **1948**, *52*(6), 1082-1089.

q. Randic, M.; Guo, X.; Oxley, T.; Krishnapriyan, H.; Naylor, L. Wiener matrix invariants. *J. Chem. Inf. Comput. Sci.*, **1994**, *34*(2), 361-367.

r. Niederfellner, J.; Lenoir, D.; Matuschek, G.; Rehfeldt, F.; Utschick, H.; Brügemann, R. Description of vapor pressures of polycyclic aromatic compounds by graph theoretical indices. *Quant. Struct.-Act. Relat.*, **1997**, *16*(1), 38-48.

s. Platt, J.R. Influence of neighbor bonds on additive bond properties in paraffins. *J. Chem. Phys.*, **1947**, *15*(6), 419-420.
